# Itaconic-Acid-Based Sustainable Poly(ester amide) Resin for Stereolithography

**DOI:** 10.1021/acs.macromol.1c02525

**Published:** 2022-04-14

**Authors:** Veronica Vetri Buratti, Alberto Sanz de Leon, Mirko Maturi, Letizia Sambri, Sergio Ignacio Molina, Mauro Comes Franchini

**Affiliations:** † Department of Industrial Chemistry “Toso Montanari”, University of Bologna, Viale Risorgimento 4, 40136 Bologna, Italy; ‡ Departamento de Ciencia de los Materiales e Ing. Metalúrgica y Química Inorgánica, IMEYMAT, Facultad de Ciencias, Universidad de Cádiz, Campus Río San Pedro, 11510 Puerto Real (Cádiz), Spain

## Abstract

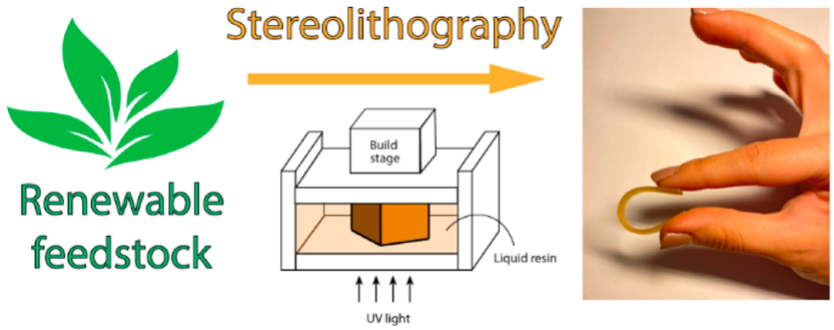

Material science is recognized as a frontrunner in achieving a sustainable future, owing to its primary reliance upon petroleum-based chemical raw materials. Several efforts are made to implement common renewable feedstocks as an alternative to common fossil resources. For this purpose, additive manufacturing (AM) represents promising and effective know-how for the replacement of high energy- and resource-demanding processes with more environmentally friendly practices. This work presents a novel biobased ink for stereolithography, which has been formulated by mixing a photocurable poly(ester amide) (PEA) obtained from renewable resources with citrate and itaconate cross-linkers and appropriate photopolymerization initiators, terminators, and dyes. The mechanical features and the relative biocompatibility of 3D-printed objects have been carefully studied to evaluate the possible resin implementation in the field of the textile fashion industry.

## Introduction

Three-dimensional (3D) printing or additive manufacturing (AM) has emerged over the past 40 years in the academic and industrial research environments as a cost- and time-effective technique for the manufacturing of customized complex objects without the use of molds and excessive waste.^1,2^ The main technological originality of AM techniques relies on the manufacturing of 3D objects by depositing the appropriate material on a substrate in a layer-by-layer fashion with the guidance of a printable command sequence achieved exploiting a digitally sliced computer-generated model created by Computer-Aided Design (CAD).^3^ Even though AM was originally developed for the rapid prototyping of tools of industrial interest, in the last years it is rapidly spreading because of its ability to print a broad array of materials, improving sustainability, design production, and costs compared to traditional methods.^4^ This technology offers indeed the possibility to reduce the cost impact of labor and materials by enabling the possibility to test the quality of a product or its parts before moving to large-scale production. For this reason, the fast-growing industrial and technological impact of AM is constantly expanding in fields such as tissue engineering, biomedical applications, jewelry, textile, dentistry, and soft robotics, depending on the material features.^5−8^

Among the different techniques known to date, stereolithography (SLA) plays a crucial role. The strategy behind its working principle relies on a liquid monomeric or oligomeric resin that is photocured and hardened when exposed to laser light, generally in the near UV range.^9^ Taking advantage of the polymerization of a photocurable ink in the presence of a photoinitiator, it is possible to obtain fine resolution complex architectures with elastomers, stretchable hydrogels, stimuli-responsive, and shape-memory materials.^10−13^ UV-mediated photopolymerization is often preferred to thermally driven radical polymerization since it enables the monomers cross-linking at or below room temperature, enabling for the employment of thermally unstable monomers and for applications that are not compatible with higher temperatures.^14^ In the last decades, the versatility of AM technologies has been proved highly suitable for applications in the textile fashion industry, allowing for the exploitation of printable raw materials for the production of fabrics and clothing.^15^ The production strategy can exploit the fabrication of an entire object in a single setup, but in some cases separately printed pieces are combined to build a more complex single garment. Polymeric materials can also be enriched by the incorporation of hard-wearing parts with comfortable textiles to allow for increased flexibility and textural properties in the final manufactured product. The supplementation of wearable smart textiles and fashion products with small, lightweight, and sensitive trackers has broadened researchers’ perspective toward the design and fabrication of e-textiles using 3D printing technology.^16,17^ Due to the availability of filaments of synthetic polyamides, polyethylene terephthalate (PET), ABS, polyvinyl acetate (PVA), cellulose composites, or polyurethanes, the main AM technology implemented for textiles production is Fused Deposition Modeling (FDM).^18^ Surprisingly, very little effort has been dedicated toward the implementation of photopolymerizable inks for textile applications.

In the last decades, the scientific community has directed a great amount of effort toward the application of renewable polymers in replacement of fossil-based ones in order to reduce the environmental impact of materials processing and production.^19^ Sustainability in the textile industry requires a significant reduction of waste generation, material transportation, and energy consumption. To date, polyesters are considered the most competitive biobased counterpart due to their unprecedented properties and affordable costs. The synthesis and characterization of flexible polyesters starting from itaconic acid and other biobased chemicals have been recently investigated by some of us.^20^ The resulting photocurable ink, obtained after the combination of the prepolymer with photopolymerization initiators/inhibitors, cross-linkers, and dyes, showed a good printability resolution in reasonable time scales, together with a total biobased content of as high as 96.5%. In the same way, several studies focused on the synthesis and application of poly(ester amide)s for additive manufacturing.^21−25^ The introduction of diamines into the synthetic pathway results in the formation of amide moieties in the polymer backbone, that can confer to the 3D printed material an improved biodegradation rate and processability, together with the desired mechanical properties.^26^ In particular, itaconic-acid-based poly(ester amide)s have not been widely investigated yet due to the possible occurrence of an aza-Michael attack of the diamine to the α,β-unsaturated double bond of the acid. A straightforward approach allows the use of presynthesized symmetric diamido-α,ω-diols as a building block, in order to prevent the activation of undesired collateral reactions.^27^

Herein, we describe the development of a fully biobased unsaturated poly(ester amide) for SLA, suitable for applications in the textile/fashion industry. Using our previous work as an outline, a long-chain fatty acid is introduced to simplify the manufacturing process, exerting a lubricating and plasticizer effect. Finally, the 3D-printed samples are thoroughly evaluated in terms of their mechanical properties and biocompatibility after the introduction of amide moieties into the polymer backbone.

## Experimental Section

All chemicals were purchased from Sigma-Aldrich Co. (St Louis, MO, USA) and used as received, except for ε-caprolactone which was freshly distilled after dehydration for 12 h over calcium hydride as a drying agent. All aqueous solutions were prepared with deionized water obtained using an ultrafiltration system (Milli-Q, Millipore) with a measured resistivity above 18 MΩ/cm.

### Synthesis of the Diamidodiol *N*,*N*′-(Butane-1,4-diyl)bis(6-hydroxyhexanamide), DAD

1,4-Butanediamine (0.225 mol, 19.88 g) was placed in a round-bottomed flask equipped with a drip funnel and under nitrogen atmosphere. Once heated to 120 °C, freshly distilled ε-caprolactone (0.451 mol, 51.3 g, 50 mL) was slowly added dropwise to the first reactant. The reaction was performed by keeping the mixture at 120 °C for 3 h. After cooling the reaction to room temperature, the white solid product was used for polymerization as-is, without further purification. The ^1^H NMR spectrum is available in Figure S1. ^1^H NMR (400 MHz, D_2_O) δ 3.60 (t, *J* = 6.6 Hz, 4H), 3.21 (d, *J* = 5.1 Hz, 4H), 2.25 (t, *J* = 7.3 Hz, 4H), 1.71–1.46 (m, 12H), 1.35 (qd, *J* = 9.7, 8.9, 6.1 Hz, 4H).

### Synthesis of the Poly(ester amide) Poly(diamidodiyl itaconate-*co*-vanillate), PEA

The poly(ester amide) (PEA) oligomer was synthesized by solventless thermal polycondensation, adapting a method already described.^28^ Briefly, itaconic acid (0.564 mol, 73.38 g) and vanillic acid (0.0564 mol, 9.49 g) were added to the previously synthesized DAD (0.226 mol, 71.38 g) and the reaction mixture was stirred at 150 °C under nitrogen flux. The mixture was gradually heated until a homogeneous melt formed, and then it was stirred for 10 h under nitrogen flow. The pressure was then reduced to approximately 0.025 atm, and the reaction was stirrred for at least 20 h. The molten product was poured in chloroform (150 mL), and the solution was then washed several times with brine and aqueous HCl solution (0.5 M). An oily yellow product was then obtained after drying the organic phase over anhydrous Na_2_SO_4_ and evaporating the solvent under vacuum. The resin was stored in chloroform solution in the fridge (+4 °C), taking care not to expose it to light. Yield = 58%, calculated as the ratio between the mass of the obtained polymer and the sum of the masses of the employed reagents subtracting the amount of condensed water under vacuum.

### Photocurable Ink Formulation and SLA 3D printing

The photocurable resin was obtained upon mixing in a round-bottomed flask 48.5 g of the oligomer PEA, 25 g of BHI, 8 g of THC, 7.5 g of lauric acid, 7.5 g of 2-hydroxyethyl methacrylate, 0.5 g of diphenyl(2,4,6-trimethyl benzoyl)phosphine oxide, 0.5 g of phenyl bis(2,4,6-trimethyl benzoyl)phosphine oxide, 0.5 g of 2,2-diethoxy acetophenone, and 2 g of 4-methoxy phenol. The mixture was magnetically stirred for 20 min to ensure the homogeneous mixing of all components. The organic solvent was then removed first by rotary evaporation and then with a high vacuum (<0.5 mmHg).

Dog-bones and bars for tensile and flexural trials have been realized via computer-assisted design (CAD), with the former matching the ISO 527-2 Type 1BA (75 × 10 × 2 mm^3^) specifications and the latter being an 80 × 10 × 4 mm^3^ rectangular bar. The virtual model was then sliced by exploiting Ultimaker Cura (4.9.1), the software provided to the costumers by the printer manufacturer. All items were printed using a Peopoly MOAI 130 SLA printer equipped with a 405 nm UV-LED laser with a spot size of 70 μm. The printer is characterized by the presence of a fluorinated ethylene-propylene (FEP) vat which is able to achieve a total building volume of 13 × 13 × 18 cm^3^ with a layer height resolution beyond 5 μm. All the optimized printing parameters are described in detail in the Supporting Information. Once the printing process is complete, the items are gently detached from the building plate and rinsed in an acetone–isopropanol solution (1:1) to remove the excess resin. Then, the raw 3D printed objects were postcured for 4 min at room temperature in a UV chamber (Sharebot CURE, Sharebot, wavelength 375–470 nm, 34.7 mW/cm^2^). For some of the printed objects, an additional postcuring treatment in a UV chamber (FormCure, Formlabs), performed for 60 min at 60 °C with a light source of 405 nm and power of 1.25 mW/cm^2^, was done.

### Characterization

^1^H and ^13^C NMR spectra were obtained on Varian Inova (14.09T, 600 MHz) and Varian Mercury (9.39 T, 400 MHz) NMR spectrometers. In all recorded spectra, chemical shifts are reported in ppm of frequency relative to the residual solvent signals for both nuclei (^1^H: 7.26 ppm for CDCl_3_ and 4.79 ppm for D_2_O; ^13^C: 77.16 ppm for CDCl_3_). ^13^C NMR analysis was performed using the ^1^H broadband decoupling mode.

Size exclusion chromatography (SEC)/gel permeation chromatography (GPC) was performed on a Knauer system (controlling a Smartline Pump 1000 equipped with a K-2301 refractive index detector). A Shimadzu Shim-Pack GPC-803 column (0.8 cm × 30 cm) and a Shimadzu Shim-Pack GPC-800P (10.0 × 4.6 mm) guard column were used as column systems. HPLC grade tetrahydrofuran (THF) was used as the eluent with a flow rate of 1 min mL^–1^. The system was calibrated with polystyrene (PS) standards obtained from PSS covering a molar mass range from 300 to 50 000 g mol^–1^ (Merck).

Differential scanning calorimetry (DSC) was performed with a Q2000 setup (TA Instruments, New Castle, DE, USA) according to the following method: (1) Heating ramp 20 °C/min, from −85 to 170 °C (1st heating); (2) ramp in cooling 20 °C/min, down to −85 °C (cooling); (3) ramp in heating 20 °C/min, from −85 to 170 °C (second heating). The *T*_g_ value was obtained from the endothermal peak in the second heating curve.

### Tensile and Flexural Tests

Tensile testing was performed in a universal testing machine (Shimadzu) at a constant speed of 1 mm/min according to ISO 527-2. Young’s modulus, tensile strength, and elongation at break values were dissected for each one of the measured specimens. Young’s modulus was determined as the slope between 0.05% and 0.5% strain in the stress–strain plots, and tensile strength was obtained as the maximum stress value in the curve. Elongation at break was obtained as the strain value at the rupture point (maximum value in the *x*-axis). In the same way, flexural testing of standardized specimens was performed in a universal testing machine (Shimadzu) at an interval of 0–5% strain at a constant speed of 1 mm/min, according to ISO 178. The flexural modulus was calculated as the slope between 0.05% and 0.25% strain. At least five specimens were tested in all cases. Results were averaged, and standard deviations were presented as error bars.

### Cell Viability Tests and Statistical Analysis

In order to assess the release of cytotoxic compounds from the 3D printed material, 1 g of the material was placed in 10 mL of cell culture medium (DMEM with 10% FBS, 1% l-Glu, 1% Pen/Strep) for 15 and 60 min, after which the solid material was removed. HaCat cells (1 × 10^4^) were seeded on polystyrene 12-wells culture plates and incubated with the cell culture medium. Treated cells and control samples were incubated at 37 °C, 5% CO_2_ for 24 h. Cell viability was assessed using a live/dead Double Staining Kit (Sigma-Aldrich). A dual fluorescent staining solution with cyto-dye and propidium iodide was added to each well according to manufacturer’s protocols. The kit utilizes cyto-dye, a permeable green fluorescent dye, to stain live cells, and propidium iodide, a nonpermeable red fluorescent dye that can only enter the cells when their membrane is damaged (dead cells). The images were acquired using a Leica DMI3000 B fluorescence microscope. All quantitative data were expressed as the mean and standard deviation. Cell count was performed using Volocity software, and data were analyzed using GraphPad Prism 6. For each analysis, statistical significance was tested using a *t* test for the selected point. *p* < 0.05 was considered statistically significant.

## Results and Discussion

### Synthesis of DAD and PEA

Among the few examples of biobased photocurable monomeric compounds, itaconic acid is a promising candidate for the production of bioderived polymers, being synthesized by distillation/dehydration of citric acid or by fermentation of biomasses by appropriately engineered bacteria.^29^ Despite it undergoing slower photopolymerization compared to commercial acrylates because of its hindered unsaturation, itaconic acid represents an economically and green alternative to petrochemical monomers.^30^ On the other hand, several synthetic routes have been designated to produce biobased aliphatic and aromatic diamines from renewable resources such as biomasses.^31^ Generally, they are mainly obtained in biotechnology via fermentation, enzymatic processes, or different engineering strategies. The biosynthesis of small amounts of 1,4-diaminobutane, also known as putrescine, occurs in healthy living cells via l-arginine or l-ornithine. Otherwise, bioengineered synthetic strategies such as gene expression, protein engineering, deletion of byproduct pathways, or enhancement of metabolic flux can be applied for the production of putrescine in *Escherichia coli* or *Corynebacterium glutamicum*. As reported in our previous work,^20^ vanillic acid, obtained by oxidation of naturally occurring vanillin, was demonstrated to play a major role in allowing for the production of biobased 3D printable inks for stereolithography. Besides imparting a pleasant vanilla smell, its light absorption properties limit of the diffusion of UV photons through the resin, allowing one to obtain high printing resolutions on the *x*–*y* plane.

The synthetic route is adapted from previous work by Papadopoulos et al., where itaconic acid was reacted under a standard polycondensation reaction with different diamines to achieve a photocurable liquid poly(ester amide) to examine its thixotropic behavior.^27^ However, the reactivity of primary amines toward the α,β-unsaturated double bond of itaconic acid was previously demonstrated to make it significantly more difficult to avoid side reactions in the formation of biobased unsaturated poly(ester amide)s. In fact, the nucleophilicity of primary amines allows for aza-Michael addition reactions to the unsaturation of itaconic acid, generating non-photocurable pyrrolidone rings. Trying to circumvent this phenomenon, we propose an innovative strategy using a symmetric diamido-α,ω-diol as the building block, starting from 1,4-diaminobutane and ε-caprolactone.^26^

**Figure 1:**
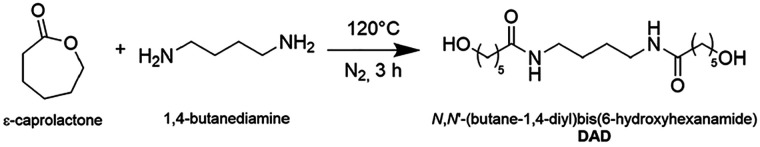
Chemical reactions for the synthesis of the diamidodiol DAD.

The synthesis of the diamidodiol (DAD, Figure 1) was conducted without the use of solvents and under dry conditions that required freshly distilled reactants.

^1^H NMR analysis reveals the absence of unreacted reagents and shows the presence of the characteristic peaks of the desired compound without byproducts (Figure S1). Thus, it was used for polymerization as-is without the need for further purifications.

**Figure 2:**
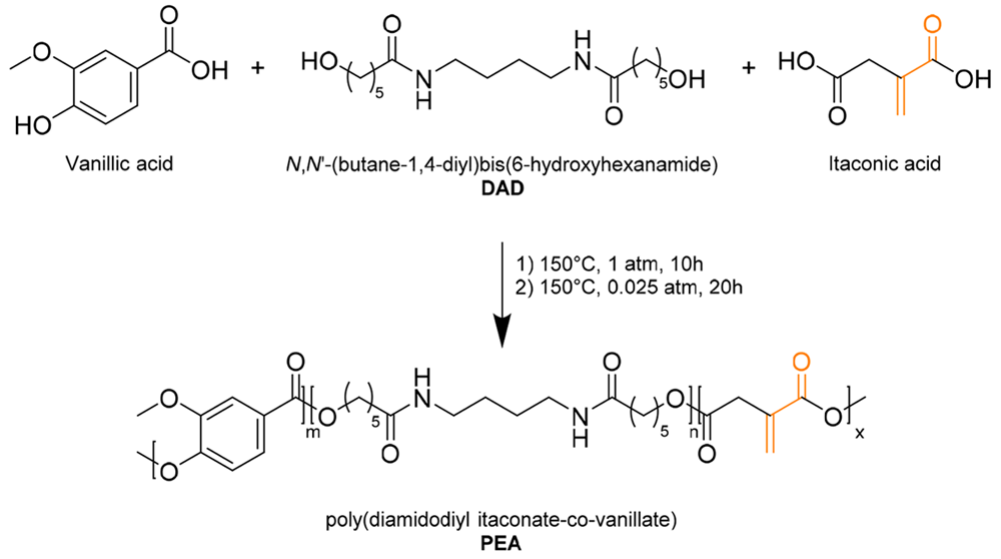
Synthetic route for the synthesis of the diamidodiol-based poly(ester amide). The photocurable group of itaconic acid is highlighted in orange.

Following the standard polycondensation synthetic procedure, the unsaturated poly(ester amide) (PEA) photocurable resin has been obtained by reacting at high temperatures itaconic acid with predetermined percentages of vanillic acid and the aforementioned DAD (Figure 2 and Table S1). From the polycondensation reaction, a fully random copolymer was formed, with itaconic and vanillic acid esterified either with the DAD or with the phenolic group of vanillic acid.

**Figure 3:**
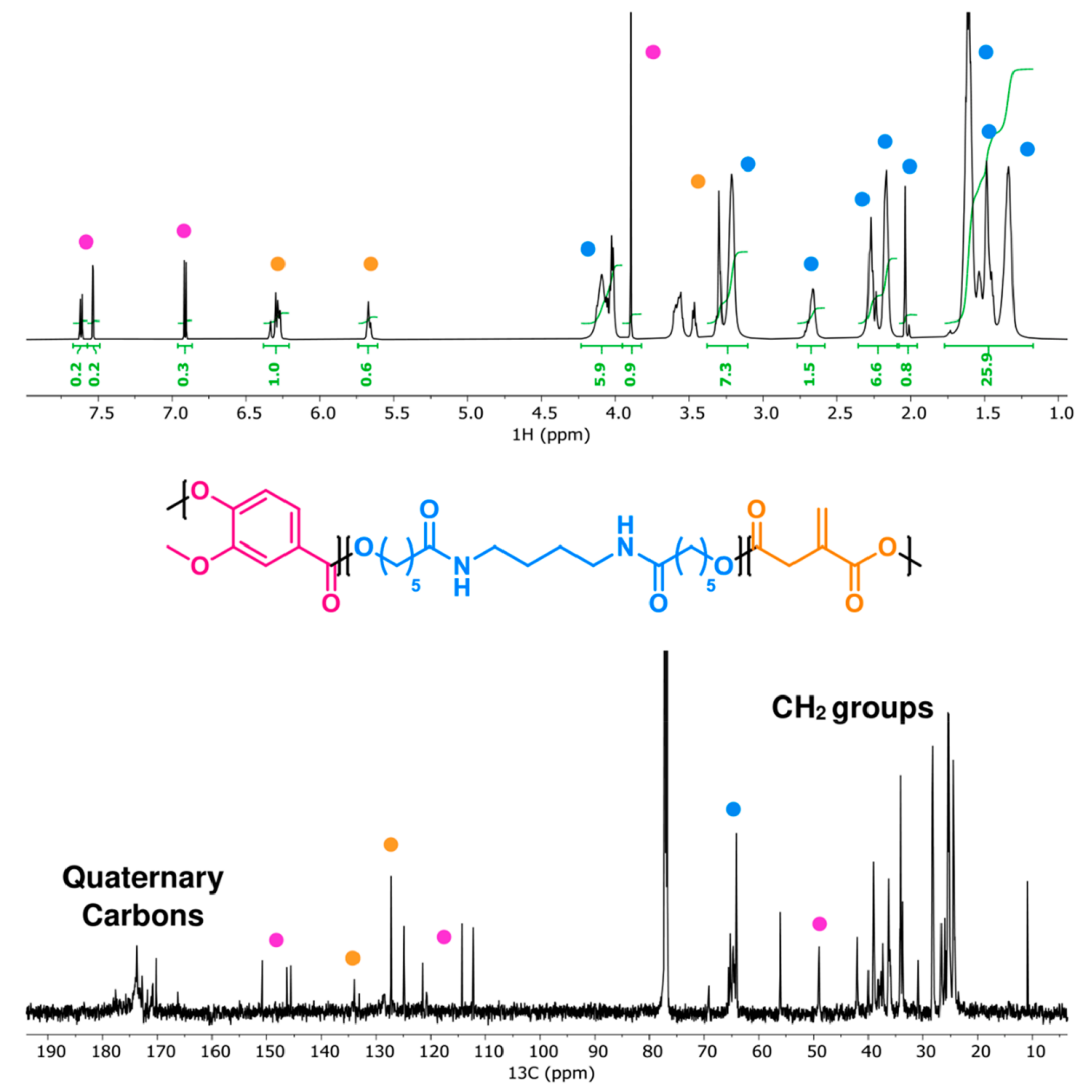
^1^H NMR (top) and ^13^C NMR (bottom) spectra (600 and 151 MHz, respectively, in CDCl_3_) of PEA, with the peak assignments to the related monomers.

The synthesized itaconic-acid-based photocurable ink was characterized by NMR spectroscopy (^1^H- and ^13^C NMR), to evaluate the outcome of the polycondensation reaction and to assess whether undesired side reactions had altered the monomers functionalities. (Figure 3). As expected, NMR analysis has revealed the full structural integrity of the employed monomers, and no signal coming from unreacted reagents was detected.

In order to evaluate weight and molar composition of the obtained polymer, NMR analysis was performed after full alkaline hydrolysis of a polymer sample. (Figure S2). The composition is reported in Table 1, whereas the whole calculation is available in the Supporting Information (SI). The notation for repeating units (*m*, *n*, and *x* in Figure 2 for vanillic acid, DAD, and itaconic acid, respectively) has been determined by setting *m* = 1 and using the molar composition to calculate the corresponding values of *m* and *n*. Following this procedure, it is possible to monitor and set the number of photopolymerizable bonds, attributable to the percentage of itaconic acid that enters the polymeric chain. As described in SI, the synthetic strategy was optimized by careful screening of the reaction conditions, to obtain mechanical features of the product in accordance with its possible applications (Table S1).

**Table 1:** Monomer Composition of the Synthesized PEA and the Corresponding Values for the Number of Repeating Units in the Average Polymer Structure, Obtained by Integration of the ^1^H NMR Spectrum of a PEA Sample after Alkaline Hydrolysis

	itaconic acid	vanillic acid	diamidodiol
molar composition	28 mol %	5 mol %	67 mol %
weight composition	14 wt %	3 wt %	84 wt %
	***x***	***m***	***n***
	5	1	12

The average molecular weight of the poly(ester amide) was determined by size exclusion chromatography (SEC)/gel permeation chromatography (GPC), revealing that for the prepared polymer *M*_n_ = 1050 g mol^–1^ and *M*_w_ = 1575 g mol^–1^, corresponding to PDI = 1.500. This low polymerization degree is in accordance with the catalyst-free thermal polycondensation selected synthetic route, allowing for the obtainment of a relatively low-viscosity liquid with rheological properties compatible with its formulation in liquid resins for stereolithography. Finally, differential scanning calorimetry (DSC) has been performed on the printed objects (Figure S3). DSC analysis of the photocured material reveals the presence of one small endothermic process around room temperature, which can be attributed to the glass-to-plastic transition of the polymeric chains in the PEA network.

### Ink Formulation

The classic composition of a resin suitable for SLA 3D printing requires the presence of different components, including the photocurable substrate with reactive acrylate/methacrylates moieties, cross-linkers, photopolymerization initiators, and terminator. Although the obtained ink is rich in photopolymerizable bonds, the latter alone is not enough to support the realization of sophisticated constructs that would collapse on themselves during the printing process. For such purposes, the formulation is enriched with some synthetic cross-linkers. Among these, bis(2-(methacryloyloxyethyl) itaconate (BHI) and tris(2-(methacryloyloxyethyl) citrate (THC) have already been extensively described in our previous work.^20^ Such esters of 2-hydroxyethyl methacrylate (HEMA) can be considered as biobased due to the renewable source of the carboxylic acids (itaconic and citric acid). As the methacrylic ester of ethylene glycol, HEMA can be considered biobased because while ethylene glycol can be produced by fermentation of xylose in *E. coli*, methacrylic acid can be manufactured by catalytic oxidative dehydrogenation of biosynthetic isobutyric acid.^32−34^

**Figure 4:**
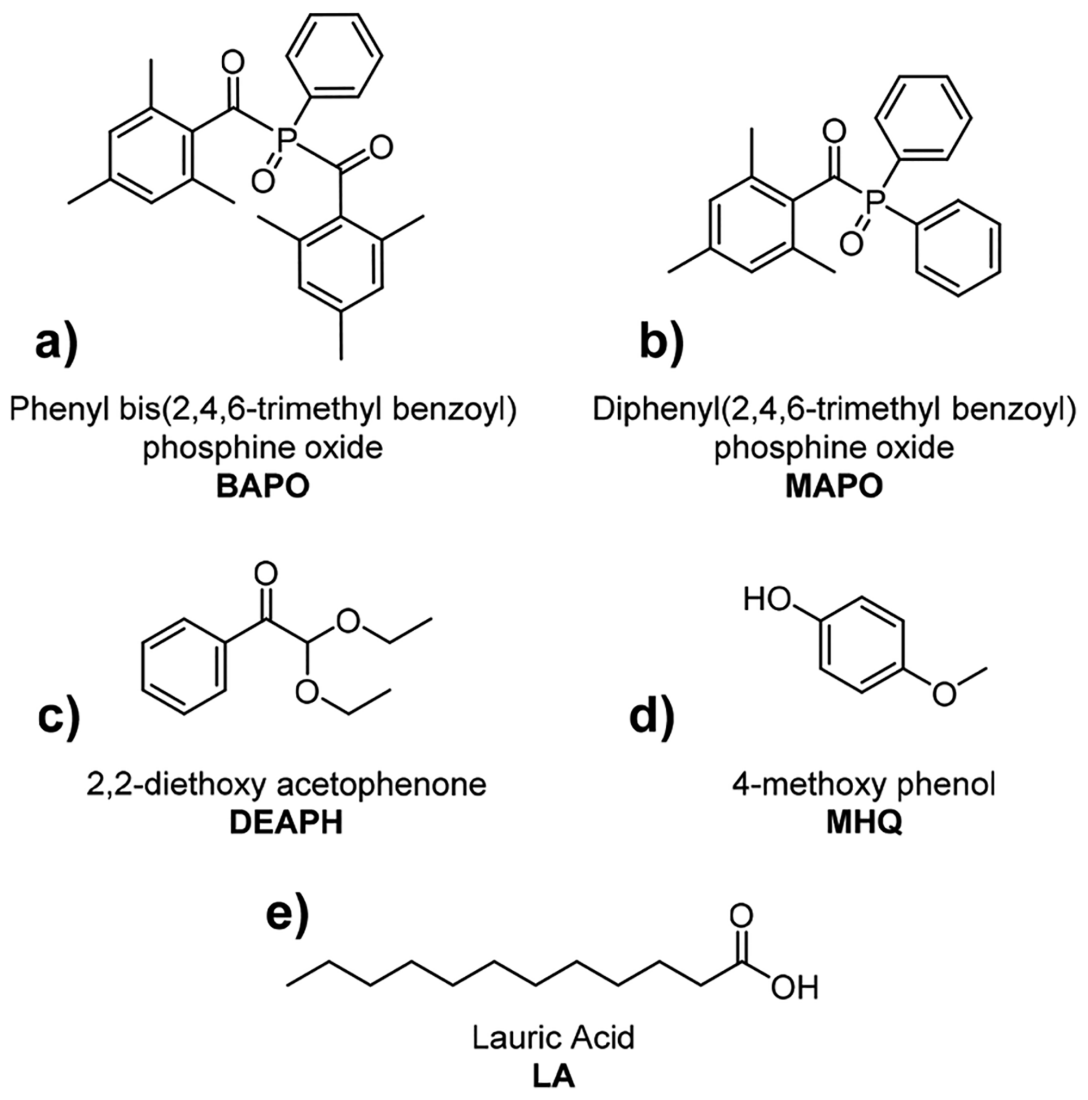
Chemical structures of the employed photoinitiators (a–c), radical terminator (d), and plasticizer (e).

The generation of reactive free radicals by the interaction of the appropriate photoinitiating system with UV light is required for the activation of the monomers in the mixture, generating larger radicals that allow for the polymer chain growth.^14^ In particular, we explored the combination of two acyl phosphine oxides such as phenyl bis(2,4,6-trimethyl benzoyl)phosphine oxide (Figure 4a, BAPO, 0.5 wt %) and diphenyl(2,4,6-trimethyl benzoyl)phosphine oxide (Figure 4b, MAPO, 0.5 wt %) together with 2,2-diethoxy acetophenone (Figure 4c, DEAPH, 0.5 wt %). Interestingly, one method to control photoinduced polymerization and to avoid the overcure phenomenon is to use radical terminators, which in our case is represented by 4-methoxy phenol (Figure 4d, MHQ, 2 wt %) that has been selected due to its low cost and its relatively high effectiveness and safety.

On the other hand, lauric acid (Figure 4e, LA) is a medium-chain fatty acid found in nature in triglycerides, being particularly abundant in coconut milk and oil, laurel, and palm kernel oil.^35^ As reported in the literature, the usage of this naturally occurring compound, in addition to the modulation of mechanical properties, has been extensively described as a lubricant and plasticizer in 3D printing by material extrusion and composite biofilm realization, due to its ability to form H-bonds.^36,37^ In this case, it is expected to efficiently lubricate and plasticize surfaces, making the process of detaching the final product from the build platform easier and avoiding fractures of the finished product. A saturated acid was deliberately chosen so as not to have possible interference with the polymerization and printing process. The effects of its addition are shown in Figure S4. LA is solid with a melting point around 43 °C, so this issue must be considered during the formulation of the printing resin. It cannot be used at high concentrations; otherwise, the risk of inducing solidification of the ink during the polymerization process inside the vessel increases. The optimized value of its concentration within the printing formulation corresponds to 7.5 wt %. Finally, the right amount of photocurable bonds in order to optimize printability and to reach the predetermined target has been achieved by adding a small amount of HEMA (7.5 wt %). The photocurable resin was obtained upon mixing in a round-bottomed flask a certain amount of the prepolymer (PEA) with the relative proportions of the other components, as reported in Table 2. This approach allows for the formulation of a renewable resin with a biobased content as high as 96.5% since all the building blocks employed for the synthesis of PEA and the cross-linkers can be considered as renewable and biobased.

**Table 2:** Weight Composition of the Developed Biobased Ink for SLA 3D Printing

photocurable ink formulation	
poly(ester amide) (PEA)	48.5 wt %
bis(2-(methacryloyloxyethyl) itaconate (BHI)	25 wt %
tris(2-(methacryloyloxyethyl) citrate (THC)	8 wt %
lauric acid (LA)	7.5 wt %
2-hydroxyethyl methacrylate (HEMA)	7.5 wt %
diphenyl(2,4,6-trimethylbenzoyl)phosphine oxide (MAPO)	0.5 wt %
phenylbis(2,4,6-trimethylbenzoyl)phosphine oxide (BAPO)	0.5 wt %
2,2-diethoxy acetophenone (DEAP)	0.5 wt %
4-methoxyphenol (MHQ)	2 wt %

### Optimization of 3D Printing Parameters

Printing parameters are optimized to reach products with good surface quality, sufficient mechanical strength, negligible dimensional error, and minimum production time. The first step involves the slicing of the virtual models that have been performed by exploiting Ultimaker Cura (4.9.1) software (Figure S5). The variation of some key parameters in the slicing phase (referred to as external, since are not machine-dependent) such as the print speed, the layer height, and the travel speed, allows optimization of the total printing process. However, the initial layer print speed must be kept under control, to ensure the correct photopolymerization of the base layers, which will be subjected to greater stress during the detachment from the building plate and which will ensure the adhesion of the resin to the build stage. The final object can be more or less compact according to the number of the top/bottom layers, inner/outer wall lines, and their relative thickness, in agreement with the final object target. In the same way, the printing speed can be tuned by varying the infill density and its relative pattern to create objects that can cover a wide range of applications. The next step foresees the optimization of the printer parameters (referred to as internal parameters), which include a first calibration of the instrument and subsequently the customization based on the type of resin. The first attempts must be aimed at identifying the minimum laser power value necessary to allow the resin photopolymerization, to ensure the maximum printing resolution. Standard printing parameters suggested by the printer manufacturer were first employed, leading to limited polymerization and incomplete 3D printing. To improve the quality of the 3D printing process, laser speed was gradually decreased and laser power and infill density were gradually increased until tensile test dog-bones were successfully formed with good resolution. The exposure to the laser must be enough to allow the object to harden and self-sustain, but at the same time too long irradiation times would lead to overcuring phenomena with a loss of resolution as a consequence. Most of the other tunable parameters are related to the movement of the printing plate toward the ink vat during the separation of two subsequent layers, in terms of the degree of inclination and tilt speed. All the optimized parameters are shown in the Supporting Information (Tables S2 and S3). However, it is very difficult to standardize this type of optimization because it is strongly dependent on the 3D printer since the laser power is not constant during the lifespan of the 3D printing machine.

### Mechanical Properties of the 3D Printed Objects

**Figure 5:**
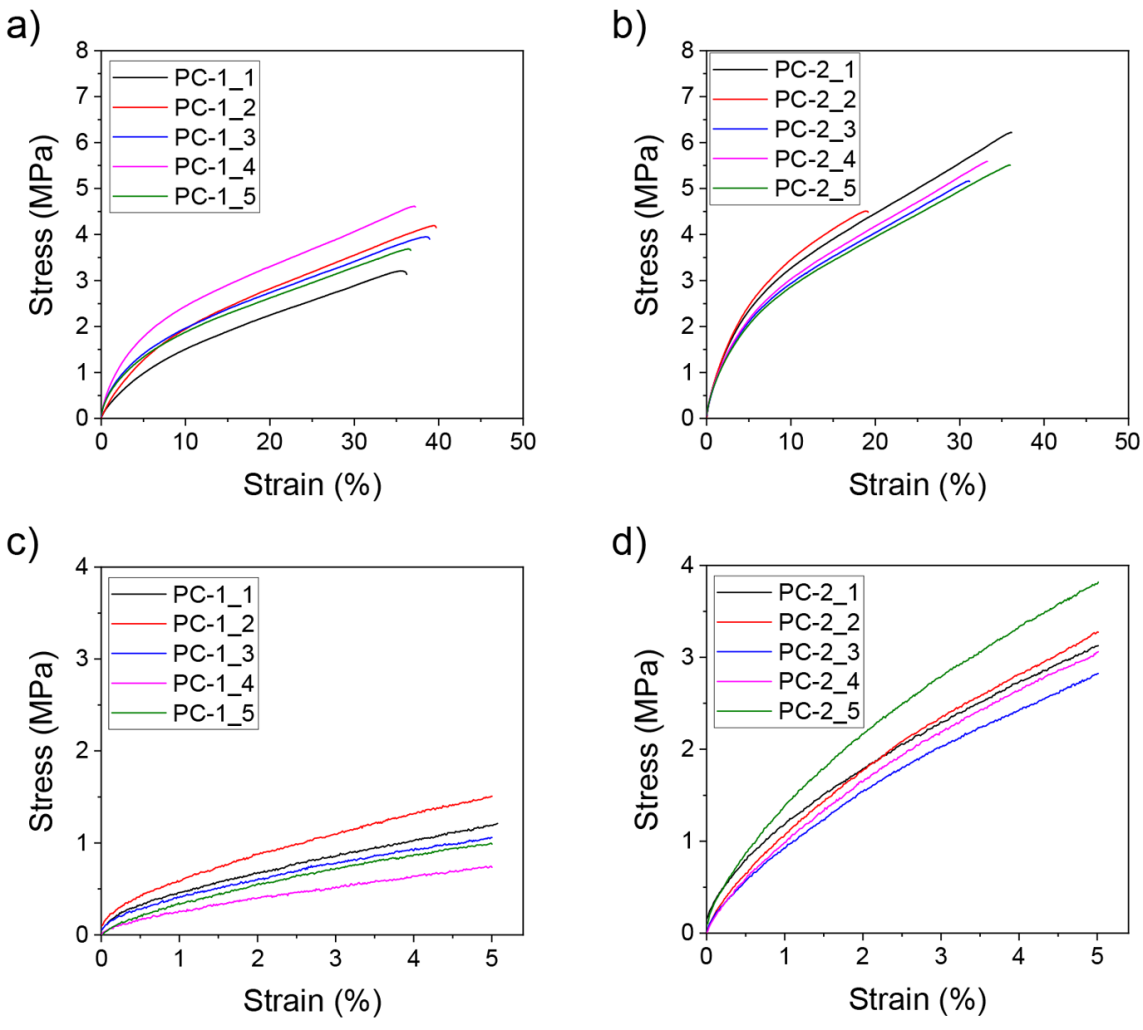
(a,b) Tensile and (c,d) flexural testing curves of 3D printed PEA structures. PC-1 samples were UV-cured for 4 min at 34.7 mW/cm^2^, while PC-2 samples were subjected to a further UV postcuring for 60 min at 60 °C at 1.25 mW/cm^2^. Five independent tests are presented for each sample.

After the optimization of the printing parameters as described in the Supporting Information, dog-bone specimens for tensile testing were printed according to ISO 527-1BA, while 80 × 10 × 4 mm^3^ rectangular bars were produced for flexural tests according to ISO 178 (Figure S6). After printing, all 3D printed specimens were first cured at room temperature for 4 min in a UV chamber equipped with a 34.7 mW/cm^2^ UV light source; then, in order to evaluate the effect of further cross-linking on the mechanical properties of the printed material, we explored the effect of a secondary postcuring process performed at higher temperature (60 °C) with lower light power (1.25 mW/cm^2^) and for longer times (60 min). Figure 5 shows the tensile and flexural strain–stress curves of five independent measurements of the PEA samples printed after UV curing for 4 min at 34.7 mW/cm^2^ (PC-1) and subsequent postprocessing for 60 min at 60 °C at 1.25 mW/cm^2^ (PC-2). Average results dissected from the 5 repeats tested for each sample are summarized in Table 3.

**Table 3:** Summary of the Mechanical Parameters Dissected from Tensile and Flexural Testing

	Young’s modulus (MPa)	tensile strength (MPa)	elongation at break (%)	flexural modulus (MPa)
PC-1	53.0 ± 17.7	3.9 ± 0.5	37.8 ± 1.4	53.5 ± 13.9
PC-2	84.7 ± 5.9	5.4 ± 0.6	34.2 ± 2.4	133.8 ± 16.9

These resins show a characteristic viscoelastic behavior with two distinct linear regions with different slopes, contrary to other polymeric materials which show first a defined elastic behavior and then a plastic deformation characterized by a plateau. Samples that underwent a longer postprocessing (PC-2) exhibit higher Young’s modulus, tensile strength, and flexural modulus, indicating that this treatment allowed further cross-linking of the material without a noticeable loss in the ductility. Regardless of the postprocessing performed, the resins exhibited elongation at break values above 30% strain. Also, the resins did not fail during the flexural testing, indicating that they are able to withstand strains of 5%. The bars recovered their initial shape after nondestructive flexural bending after ca. 30 min, indicating that this material might be suitable for memory shape applications. Nonstandard, longer flexural experiments applying strains above 5% were done, and it was found that the resin breaks at strain above 15%, indicating high flexibility. These experimental pieces of evidence show values that are completely in line with the current trend and with mechanical properties of the latest generation biobased resins, for which Young’s modulus is in the range of 3.5 and 40 MPa.^38,39^ Moreover, Young’s and flexural moduli are in line with the requirements for the application of the described PEA resin for the production of textiles.

### Cell Viability Tests

**Figure 6:**
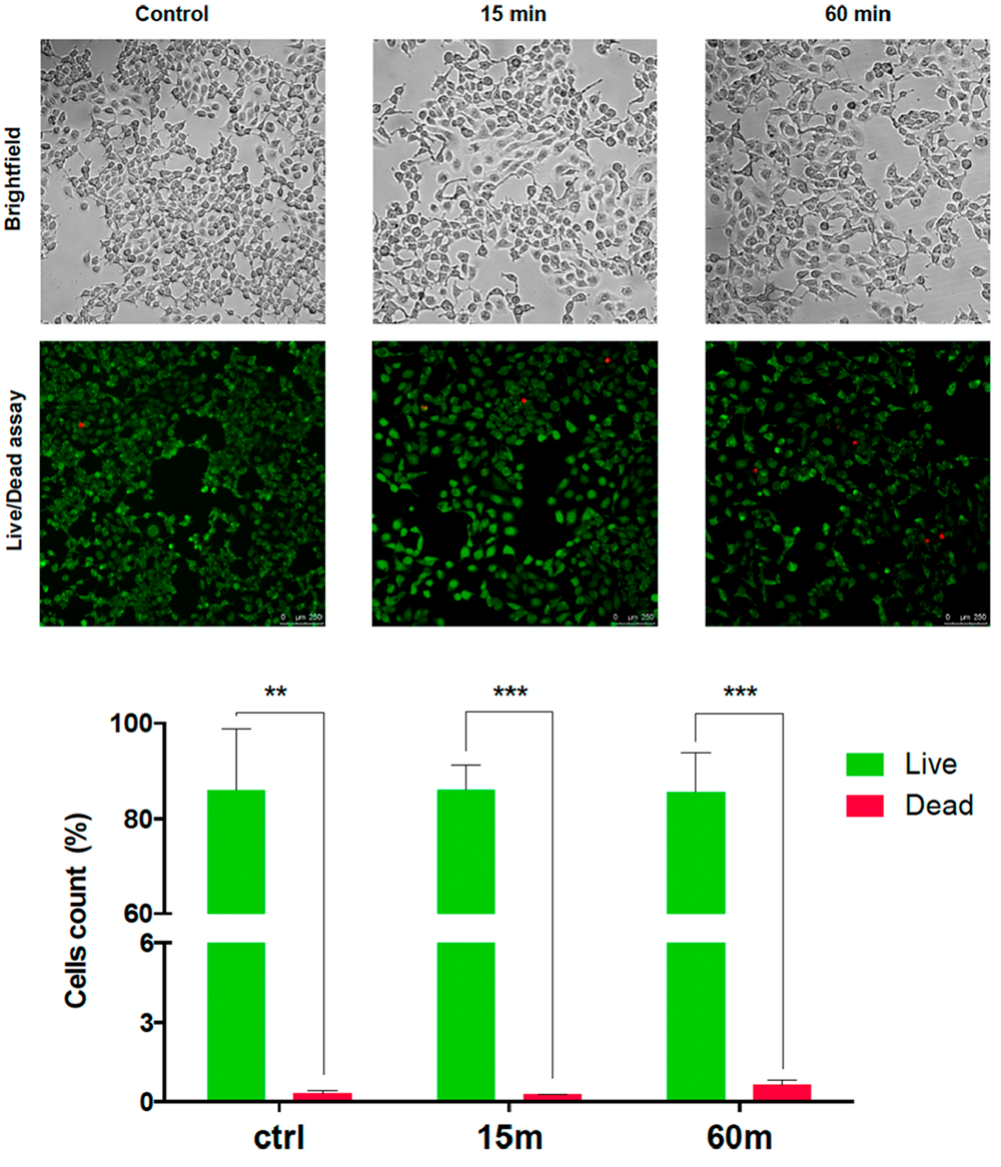
Fluorescence microscopy representative images and relative quantifies of live/dead assay (green/red) on HaCat cells seeded with and without exposure to the printed sample eluate, plotted as a result of four representative fields. **p < 0.0074; ***p < 0.0004.

In order to preliminarily assess the biocompatibility of the printed resin for possible future textile applications, an in vitro analysis of cell viability was carried out. Human keratinocytes seeded on polystyrene Petri dishes and exposed for 24 h to the eluate did not display any difference in terms of adhesion, morphology, and viability compared to the control experiment (Figure 6), demonstrating no intrinsic cytotoxicity of the reported material.

## Conclusions

In the present work, a photopolymer resin based on biobased raw materials is prepared for application in stereolithographic 3D printing. The proposed ink offers a reliable green and low-cost alternative to fossil-based formulations available on the market. Straightforward application of the formulation in a commercial SLA printer is demonstrated by the successful fabrication of tensile and flexural bars, on which a careful mechanical characterization was evaluated. The manufactured prototypes demonstrate an elastic modulus and elongation at break comparable to those of recently published biobased resins, which suggests good applicability in the identified target. In addition, samples that underwent a longer postprocessing time exhibit even more encouraging data, indicating that this treatment allowed further cross-linking of the material without affecting their performance. Cellular viability on human keratinocytes has been assessed and confirmed that no cytotoxicity response is revealed. Taken together, the last two features suggest a possible future focus on the development of stereolithography-based formulation of green textiles that would allow the employment of the reported formulation for the manufacturing of fashion products and accessories.

## Supporting Information Available

Additional ^1^H NMR spectra, compositional analysis; thermal analysis of 3D printed materials and instrumental parameters for stereolithography; and tensile and flexural testing curves (PDF)

## References

[ref1] Jasiuk I.; Abueidda D. W.; Kozuch C.; Pang S.; Su F. Y.; McKittrick J. An Overview on Additive Manufacturing of Polymers. JOM 2018, 70 (3), 275–283. 10.1007/s11837-017-2730-y.

[ref2] Gibson I.; Rosen D. W.; Stucker B. Additive Manufacturing Technologies; Springer, 2010.

[ref3] Ligon S. C.; Liska R.; Stampfl J.; Gurr M.; Mülhaupt R. Polymers for 3D Printing and Customized Additive Manufacturing. Chem. Rev. 2017, 117 (15), 10212–10290. 10.1021/acs.chemrev.7b00074. 28756658 PMC5553103

[ref4] Kianian B. 3D Printing and Additive Manufacturing State of the Industry, Annual Worldwide Progress Report: Chapters Titles: The Middle East, and Other Countries. In Wohlers Report - 3D Printing and Additive Manufacturing State of the Industry; Wohlers Associates, Inc., 2017.

[ref5] Diez-Escudero A.; Andersson B.; Persson C.; Hailer N. P. Hexagonal Pore Geometry and the Presence of Hydroxyapatite Enhance Deposition of Mineralized Bone Matrix on Additively Manufactured Polylactic Acid Scaffolds. Mater. Sci. Eng., C 2021, 125, 112091 10.1016/j.msec.2021.112091. 33965101

[ref6] Klotz U. E.; Tiberto D.; Held F. Optimization of 18-Karat Yellow Gold Alloys for the Additive Manufacturing of Jewelry and Watch Parts. Gold Bull. 2017, 50 (2), 111–121. 10.1007/s13404-017-0201-4.

[ref7] Morrow J.; Hemleben S.; Menguc Y. Directly Fabricating Soft Robotic Actuators With an Open-Source 3-D Printer. IEEE Robot. Autom. Lett. 2017, 2 (1), 277–281. 10.1109/LRA.2016.2598601.

[ref8] Anssari Moin D.; Derksen W.; Verweij J. P.; van Merkesteyn R.; Wismeijer D. A Novel Approach for Computer-Assisted Template-Guided Autotransplantation of Teeth With Custom 3D Designed/Printed Surgical Tooling. An Ex Vivo Proof of Concept. J. Oral Maxillofac. Surg. 2016, 74 (5), 895–902. 10.1016/j.joms.2016.01.033. 26907556

[ref9] Bagheri A.; Jin J. Photopolymerization in 3D Printing. ACS Appl. Polym. Mater. 2019, 1 (4), 593–611. 10.1021/acsapm.8b00165.

[ref10] Melhem M. R.; Park J.; Knapp L.; Reinkensmeyer L.; Cvetkovic C.; Flewellyn J.; Lee M. K.; Jensen T. W.; Bashir R.; Kong H.; et al. 3D Printed Stem-Cell-Laden, Microchanneled Hydrogel Patch for the Enhanced Release of Cell-Secreting Factors and Treatment of Myocardial Infarctions. ACS Biomater. Sci. Eng. 2017, 3 (9), 1980–1987. 10.1021/acsbiomaterials.6b00176. 33440553

[ref11] Zarek M.; Layani M.; Cooperstein I.; Sachyani E.; Cohn D.; Magdassi S. 3D Printing of Shape Memory Polymers for Flexible Electronic Devices. Adv. Mater. 2016, 28 (22), 4449–4454. 10.1002/adma.201503132. 26402320

[ref12] Zhao T.; Yu R.; Li X.; Cheng B.; Zhang Y.; Yang X.; Zhao X.; Zhao Y.; Huang W. 4D Printing of Shape Memory Polyurethane via Stereolithography. Eur. Polym. J. 2018, 101, 120–126. 10.1016/j.eurpolymj.2018.02.021.

[ref13] Woodward D. I.; Purssell C. P.; Billson D. R.; Hutchins D. A.; Leigh S. J. Additively-Manufactured Piezoelectric Devices. Phys. status solidi 2015, 212 (10), 2107–2113. 10.1002/pssa.201532272.

[ref14] Bird D.; Caravaca E.; Laquidara J.; Luhmann K.; Ravindra N. M. Formulation of Curable Resins Utilized in Stereolithography; Springer, 2019.

[ref15] Chakraborty S.; Biswas M. C. 3D Printing Technology of Polymer-Fiber Composites in Textile and Fashion Industry: A Potential Roadmap of Concept to Consumer. Compos. Struct. 2020, 248, 112562 10.1016/j.compstruct.2020.112562.

[ref16] Wei S.; Qu G.; Luo G.; Huang Y.; Zhang H.; Zhou X.; Wang L.; Liu Z.; Kong T. Scalable and Automated Fabrication of Conductive Tough-Hydrogel Microfibers with Ultrastretchability, 3D Printability, and Stress Sensitivity. ACS Appl. Mater. Interfaces 2018, 10 (13), 11204–11212. 10.1021/acsami.8b00379. 29504395

[ref17] Yin X.-Y.; Zhang Y.; Cai X.; Guo Q.; Yang J.; Wang Z. L. 3D Printing of Ionic Conductors for High-Sensitivity Wearable Sensors. Mater. Horizons 2019, 6 (4), 767–780. 10.1039/C8MH01398E.

[ref18] Chatterjee K.; Ghosh T. K. 3D Printing of Textiles: Potential Roadmap to Printing with Fibers. Adv. Mater. 2020, 32 (4), 1902086 10.1002/adma.201902086. 31788860

[ref19] Voet V. S. D.; Strating T.; Schnelting G. H. M.; Dijkstra P.; Tietema M.; Xu J.; Woortman A. J. J.; Loos K.; Jager J.; Folkersma R. Biobased Acrylate Photocurable Resin Formulation for Stereolithography 3D Printing. ACS Omega 2018, 3 (2), 1403–1408. 10.1021/acsomega.7b01648. 31458469 PMC6641428

[ref20] Maturi M.; Pulignani C.; Locatelli E.; Vetri Buratti V.; Tortorella S.; Sambri L.; Comes Franchini M. Phosphorescent Bio-Based Resin for Digital Light Processing (DLP) 3D-Printing. Green Chem. 2020, 22 (18), 6212–6224. 10.1039/D0GC01983F.

[ref21] Díaz A.; Katsarava R.; Puiggalí J. Synthesis, Properties and Applications of Biodegradable Polymers Derived from Diols and Dicarboxylic Acids: From Polyesters to Poly(Ester Amide)S. Int. J. Mol. Sci. 2014, 15 (5), 7064–7123. 10.3390/ijms15057064. 24776758 PMC4057662

[ref22] Ruano G.; Díaz A.; Tononi J.; Torras J.; Puiggalí J.; Alemán C. Biohydrogel from Unsaturated Polyesteramide: Synthesis, Properties and Utilization as Electrolytic Medium for Electrochemical Supercapacitors. Polym. Test. 2020, 82, 106300 10.1016/j.polymertesting.2019.106300.

[ref23] Ruano G.; Tononi J.; Curcó D.; Puiggalí J.; Torras J.; Alemán C. Doped Photo-Crosslinked Polyesteramide Hydrogels as Solid Electrolytes for Supercapacitors. Soft Matter 2020, 16 (34), 8033–8046. 10.1039/D0SM00599A. 32785400

[ref24] Macías S. I.; Ruano G.; Borràs N.; Alemán C.; Armelin E. UV assisted photo reactive polyether-polyesteramide resin for future applications in 3D printing. J. Polym. Sci. 2022, 60 (4), 688–700. 10.1002/pol.20210626.

[ref25] Ansari V.; Calore A.; Zonderland J.; Harings J. A. W.; Moroni L.; Bernaerts K. V. Additive Manufacturing of α-Amino Acid Based Poly(Ester Amide)s for Biomedical Applications. Biomacromolecules 2022, 23 (3), 1083–1100. 10.1021/acs.biomac.1c01417. 35050596 PMC8924872

[ref26] Garg P.; Keul H.; Klee D.; Möller M. Concept and Synthesis of Poly(Ester Amide)s with One Isolated, Two or Three Consecutive Amide Bonds Randomly Distributed Along the Polyester Backbone. Des. Monomers Polym. 2009, 12 (5), 405–424. 10.1163/138577209X12486896623454.

[ref27] Papadopoulos L.; Kluge M.; Bikiaris D. N.; Robert T. Straightforward Synthetic Protocol to Bio-Based Unsaturated Poly(Ester Amide)s from Itaconic Acid with Thixotropic Behavior. Polymers (Basel). 2020, 12 (4), 980 10.3390/polym12040980. 32331487 PMC7240367

[ref28] Barrett D. G.; Merkel T. J.; Luft J. C.; Yousaf M. N. One-Step Syntheses of Photocurable Polyesters Based on a Renewable Resource. Macromolecules 2010, 43 (23), 9660–9667. 10.1021/ma1015424.

[ref29] Kumar S.; Krishnan S.; Samal S. K.; Mohanty S.; Nayak S. K. Itaconic Acid Used as a Versatile Building Block for the Synthesis of Renewable Resource-Based Resins and Polyesters for Future Prospective: A Review. Polym. Int. 2017, 66 (10), 1349–1363. 10.1002/pi.5399.

[ref30] Pérocheau Arnaud S.; Malitowski N. M.; Meza Casamayor K.; Robert T. Itaconic Acid-Based Reactive Diluents for Renewable and Acrylate-Free UV-Curing Additive Manufacturing Materials. ACS Sustain. Chem. Eng. 2021, 9 (50), 17142–17151. 10.1021/acssuschemeng.1c06713.

[ref31] Wang X.; Gao S.; Wang J.; Xu S.; Li H.; Chen K.; Ouyang P. The Production of Biobased Diamines from Renewable Carbon Sources: Current Advances and Perspectives. Chin. J. Chem. Eng. 2021, 30, 4–13. 10.1016/j.cjche.2020.12.009.

[ref32] Pereira B.; Zhang H.; De Mey M.; Lim C. G.; Li Z. J.; Stephanopoulos G. Engineering a Novel Biosynthetic Pathway in Escherichia Coli for Production of Renewable Ethylene Glycol. Biotechnol. Bioeng. 2016, 113 (2), 376–383. 10.1002/bit.25717. 26221864

[ref33] Wang Y.; Xian M.; Feng X.; Liu M.; Zhao G. Biosynthesis of Ethylene Glycol from D-Xylose in Recombinant Escherichia Coli. Bioengineered 2018, 9 (1), 233–241. 10.1080/21655979.2018.1478489. 29865993 PMC6984763

[ref34] Le Nôtre J.; Witte-van Dijk S. C. M.; van Haveren J.; Scott E. L.; Sanders J. P. M. Synthesis of Bio-Based Methacrylic Acid by Decarboxylation of Itaconic Acid and Citric Acid Catalyzed by Solid Transition-Metal Catalysts. ChemSusChem 2014, 7 (9), 2712–2720. 10.1002/cssc.201402117. 25045161

[ref35] Dayrit F. M. The Properties of Lauric Acid and Their Significance in Coconut Oil. J. Am. Oil Chem. Soc. 2015, 92 (1), 1–15. 10.1007/s11746-014-2562-7.

[ref36] Adorna J. A.; Aleman C. K. A.; Gonzaga I. L. E.; Pangasinan J. N.; Sisican K. M. D.; Dang V. D.; Doong R.-A.; Ventura R. L. G.; Ventura J.-R. S. Effect of Lauric Acid on the Thermal and Mechanical Properties of Polyhydroxybutyrate (PHB)/Starch Composite Biofilms. Int. J. Polym. Sci. 2020, 2020, 1–11. 10.1155/2020/7947019.

[ref37] Chaunier L.; Guessasma S.; Belhabib S.; Della Valle G.; Lourdin D.; Leroy E. Material Extrusion of Plant Biopolymers: Opportunities & Challenges for 3D Printing. Addit. Manuf. 2018, 21, 220–233. 10.1016/j.addma.2018.03.016.

[ref38] Meiorin C.; Aranguren M. I.; Mosiewicki M. A. Smart and Structural Thermosets from the Cationic Copolymerization of a Vegetable Oil. J. Appl. Polym. Sci. 2011, 5071–5078. 10.1002/app.35630.

[ref39] Wu S.; Luo M.; Darensbourg D. J.; Zeng D.; Yao Y.; Zuo X.; Hu X.; Tan D. Non-Isocyanate and Catalyst-Free Synthesis of a Recyclable Polythiourethane with Cyclic Structure. ACS Sustain. Chem. Eng. 2020, 8 (14), 5693–5703. 10.1021/acssuschemeng.0c00435.

