# Supplementary material for: Itaconic-Acid-Based Sustainable Poly(ester amide) Resin for Stereolithography
Source: Macromolecules. 2022 Apr 14;55(8):3087–95. doi: 10.1021/acs.macromol.1c02525 (PMC9937558; doi:10.1021/acs.macromol.1c02525)
Supplement: Supplementary file 1 — ma1c02525_si_001.pdf [file ma1c02525_si_001.pdf]

Itaconic Acid-based Sustainable Poly(ester-amide) Resin for Stereolithography

Veronica Vetri Buratti,[a#] Alberto Sanz de Leon,[b#] Mirko Maturi,[a] Letizia Sambri,[a] Sergio Ignacio Molina,[b] Mauro Comes Franchini[a]**

[a] Department of Industrial Chemistry "Toso Montanari", University of Bologna, Viale Risorgimento 4, 40136 Bologna, Italy.
[b] Departamento de Ciencia de los Materiales e Ing. Metalúrgica y Química Inorgánica, IMEYMAT, Facultad de Ciencias, Campus Río San Pedro, 11510 Puerto Real (Cádiz), Spain

E-mail: sergio.molina@uca.es; mauro.comesfranchini@unibo.it

SUPPORTING INFORMATION

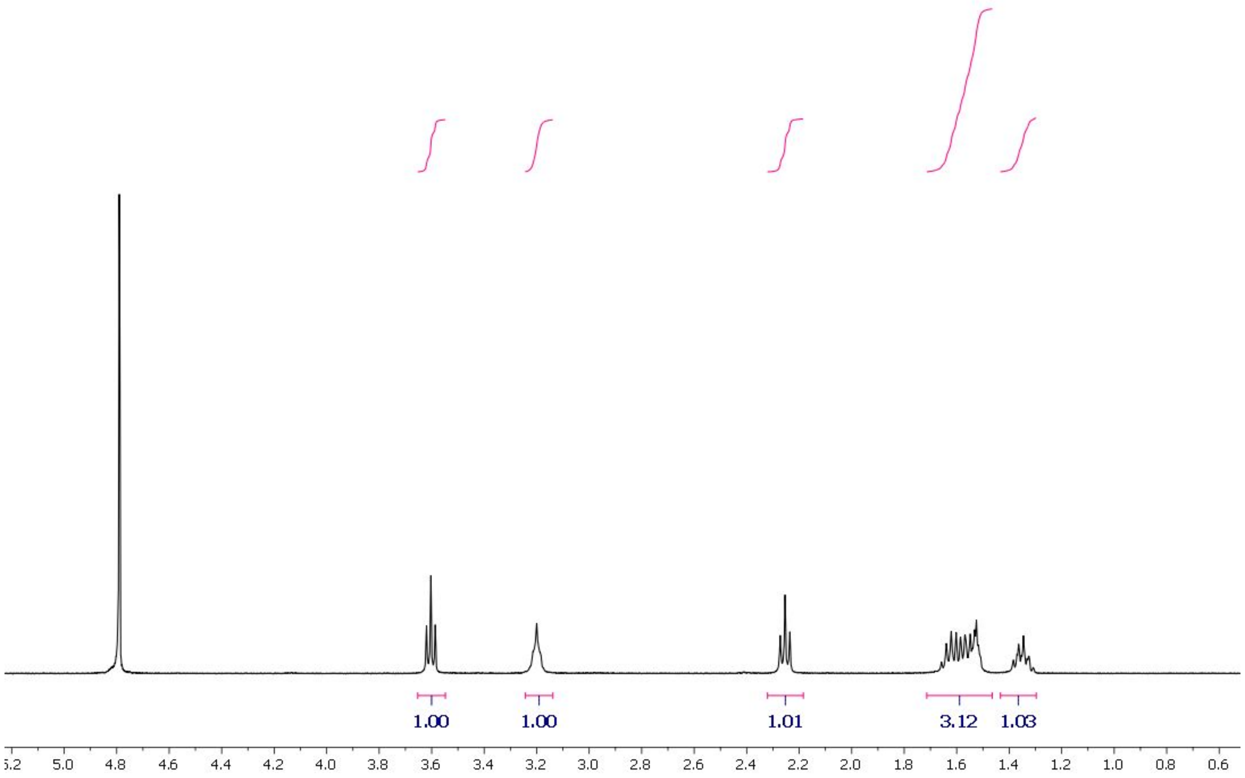

Figure S1 - ^{1}H-NMR (400 MHz, D_2O) of DAD.

PHOTOCURABLE POLY(ESTER AMIDE) – COMPOSITIONAL ANALYSIS

The ideal ink should contain a minimum number of light-susceptible bonds which allows a good printability and gives to the printed objects a good resistance. At the same time, however, they should not be in excess as they would result in extremely brittle objects. In this regard, the same polycondensation reaction was tested by increasing the amount of itaconic acid present in the reaction mixture, until the desired product was obtained (**Table S1**). The molar ratios between monomers, in particular the amount of photocurable bonds reliable to itaconic acid, were monitored by NMR spectroscopy of fully hydrolized samples. Strongly alkaline conditions are required for the complete hydrolysis of the resin, such as NaOH 3 M in deuterium oxide and high working temperatures (around 80 °C). In this way it is possible to generate simpler NMR spectra which display the typical signals of each employed monomer. Unambiguously assignable peaks have been integrated in order to establish the molar composition of the prepared oligomer.

		Itaconic Acid	Vanillic Acid	Diamidodiol (DAD)
Reaction mixture composition (equivalents)	A	1	0.2	0.8
	B	2	0.2	0.8
	C	2.5	0.2	0.8

Table S1- Different reaction conditions that have been explored. The best results were obtained with the conditions **B**.

In particular to get the molar ratio between monomers, first of all each signal integral has been divided by the number of protons it represents, and signals from the same monomers have been averaged. As a general formula:

$$n_x = \frac{1}{k_x} \sum_{m=1}^{k} \frac{\int S_m(x)}{N_{S_m(x)}}$$

Where n_x is proportional to the number of moles of monomer x, k_x is the number of signals that can be attributed to monomer x, $\int S_m(x)$ is the integral of the mth NMR signal of monomer x and $N_{S_m(x)}$ is the number of protons represented by the mth NMR signal of monomer x.

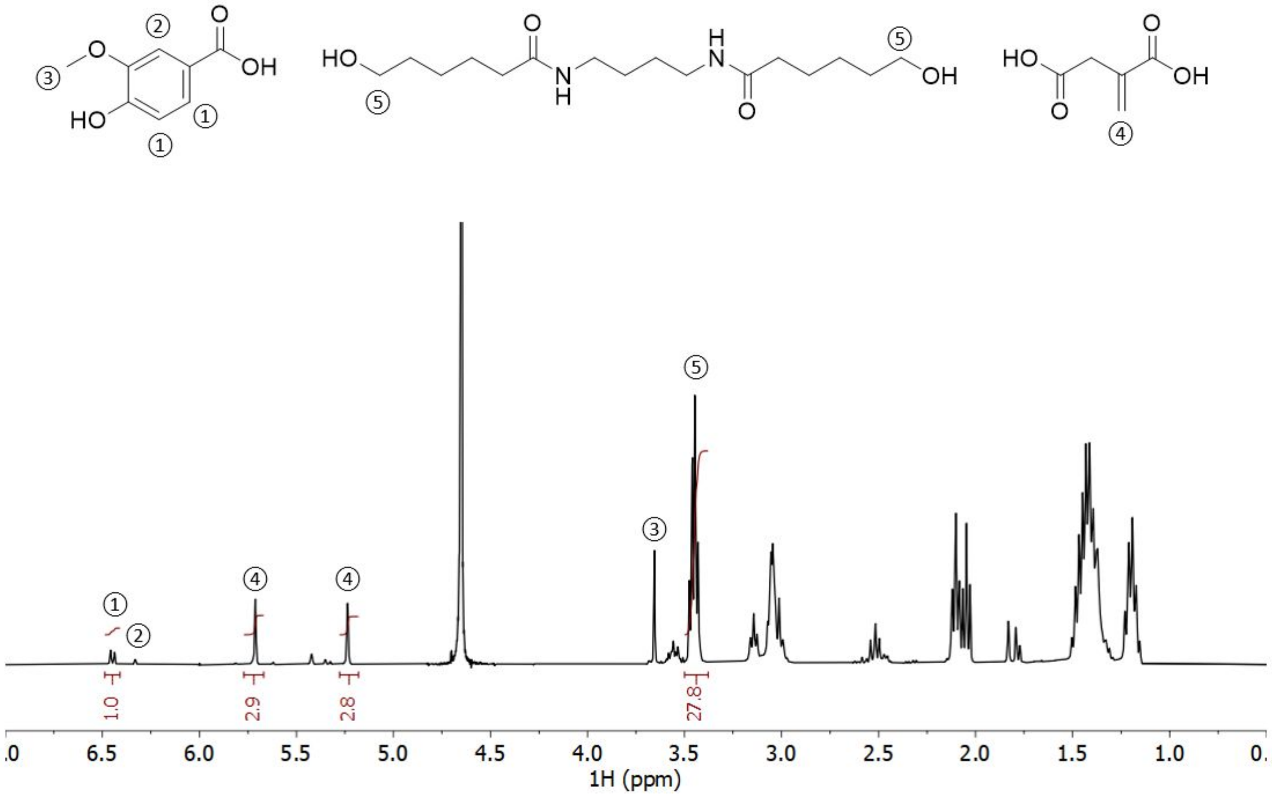

Figure S2 - ^{1}H-NMR (400 MHz, D_2O) of PEA (B) after alkaline hydrolysis with the corresponding attribution of the peaks employed for monomer quantification.

$$n_{Vanillic} = \left[\frac{1.0}{2}\right] = 0.5$$

$$n_{Itaconic} = \frac{1}{2}\left[\frac{2.9}{1} + \frac{2.8}{1}\right] = 2.85$$

$$n_{DAD} = \left[\frac{27.8}{4}\right] = 6.95$$

$$n_{Total} = \sum n_x = 10.3$$

And the molar ratios calculated accordingly:

$$\boldsymbol{x_{Vanillic} = 5.0\ mol\%}$$

$$\boldsymbol{x_{Itaconic} = 28\ mol\%}$$

$$\boldsymbol{x_{DAD} = 67\ mol\%}$$

Then, the weight composition has been obtained by using the same approach, but multiplying each n_x by the corresponding molecular weight:

$$\left.\begin{array}{l} m_{Vanillic} = n_{Vanillic} \cdot MW_{Vanillic} = 0.5 \cdot 168.14 = 84.07 \\ m_{Itaconic} = n_{Itaconic} \cdot MW_{Itaconic} = 2.85 \cdot 130.1 = 370.79 \\ m_{DAD} = n_{DAD} \cdot MW_{DAD} = 6.95 \cdot 316.14 = 2197.2 \end{array}\right\} \quad m_{Total} = \sum m_x = 2634$$

And the weight ratios calculated accordingly:

$$\boldsymbol{x_{Vanillic} = 3.3\ mol\%}$$

$$\boldsymbol{x_{Itaconic} = 14.1\ mol\%}$$

$$\boldsymbol{x_{DAD} = 83.6\ mol\%}$$

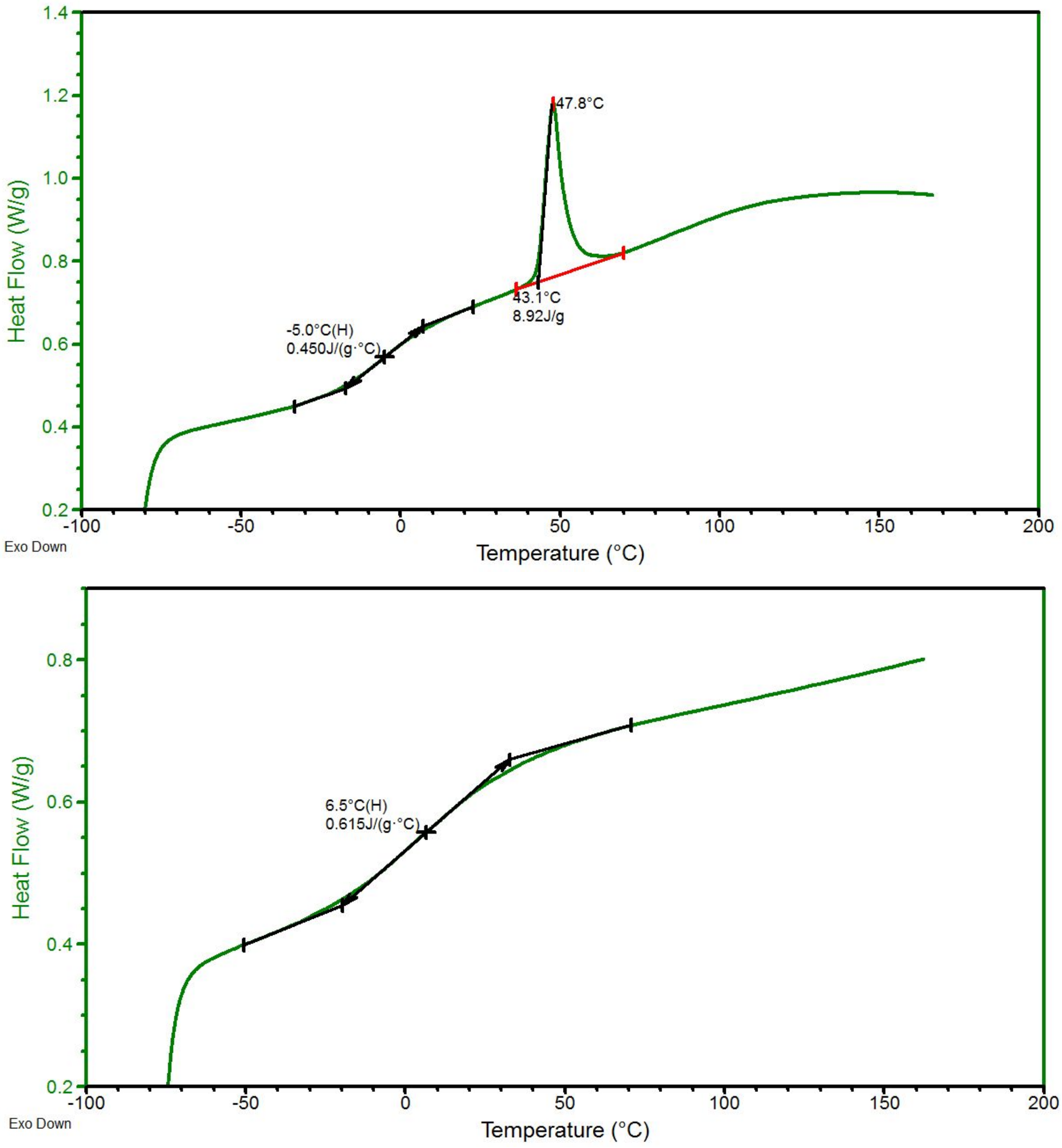

Figure S3 – Differential Scanning Calorimetry (DSC) traces of the 3D-Printed resin, obtained by heating the sample from −85 °C to +170 °C with 20 °C min^{-1} heating rate. Top: first heating; Bottom: second heating. The peak in the first heating curve is related to the melting of lauric acid.

Without lauric acid ***With lauric acid***

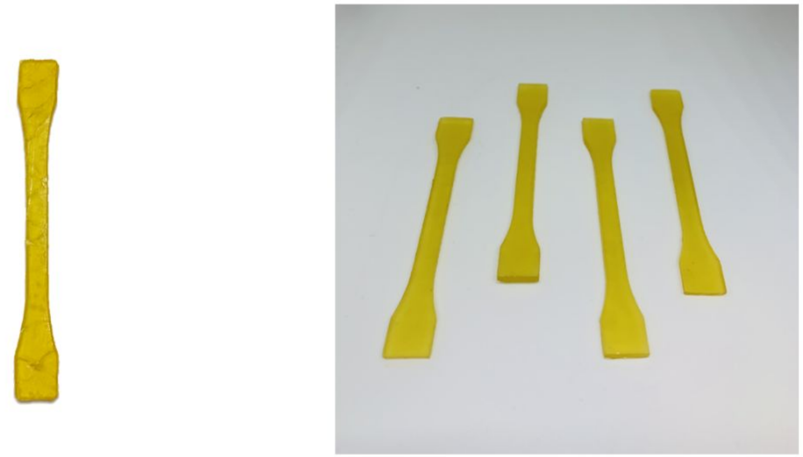

Figure S4: Effect of lauric acid on the 3D printing properties of the photocurable ink. In the absence of lauric acid, the printed object generally breaks during the detachment from the building platform and resolution is very low.

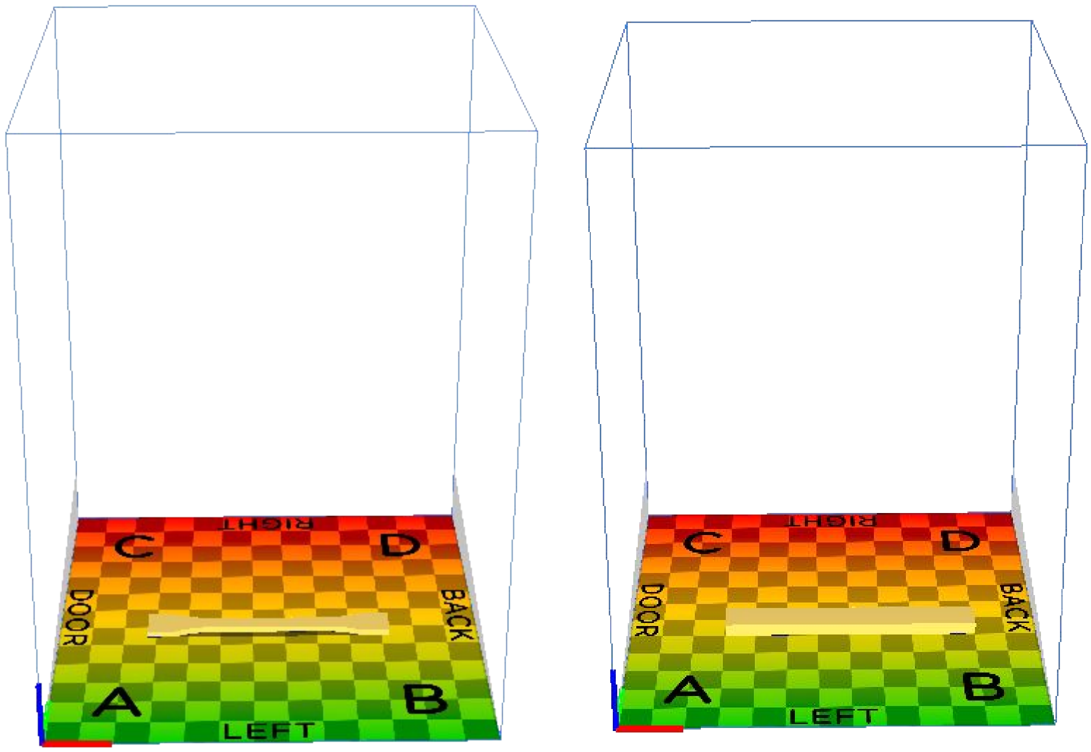

Figure S5 - Design processing of the 3D printed models using Ultimaker Cura (4.9.1) software a) Dog-bones for tensile tests (ISO-1BA, 75x10x2 mm) Printing Time 58 min; b) bars for flexural and impact tests (80x10x4 mm) Printing Time 2h 50 min.

Quality		Infill	
Layer Height	0,1 mm	Infill Density	50
Initial Layer Height	0,067 mm	Infill Line Distance	0,134
Line Widht	0,067 mm	Infill Pattern	Gyroid
Wall Line Widht	0,067 mm	Extra Infill Wall Count	0
Outer Wall Line Widht	0,067 mm	Infill Overlap percentage	15
Inner Wall Line Widht	0,067 mm	Infill Overlap	0,0101
Infill Line Widht	0,067 mm	Infill Layer Thickness	0,1
**Speed**		**Walls**	
Print Speed	30	Wall Tickhness	0.5
Infill Speed	40	Wall Line Count	2
Wall Speed	30	**Top/Bottom**	
Outer Wall Speed	20	Top/Bottom Thickness	0.1
Inner Wall Speed	20	Top Thickness	0.1
Travel Speed	50	Top Layers	2
Initial Layer Speed	15	Bottom Thickness	0.1
		Bottom Layers	0

Table S2 - Optimized external parameters for minimizing the printing time while still maintaining good resolution and structural stability.

	Optimized Value	Description	Influence on the printing process
**Z moto speed**	2	Travel speed of the building platform along the Z axis.	Increasing this value, the printing speed is improved but the greater force applied to separate the layers could cause the breakage of the object.
**PM moto speed**	12	Vat tilt speed between printing two layers.	If increased, the force applied for the separation between different layers increases and the object could break.
**Laser power**	200	Parameter that indicates the laser power.	If increased, it facilitates the hardening of the resin but reduces the resolution, due to the higher spot size.
**XY speed set**	1	Laser irradiation speed galvo.	If increased, faster printing speed but the construction of the object is not guaranteed.
**Z reset position**	1805	Vat position along the Z axis.	The right calibration allows to position the building platform perfectly on the resin surface, guaranteeing an optimal vertical construction of the object.
**PM reset position**	160	Vat inclination during the separation of the layers.	If increased, the force applied for the separation between different layers increases and the object could be damaged .
**Z follows**	2	Building plate inclination in the same direction of the tay.	When increased, it reduces the force applied for the separation of the layers, facilitating the formation of the desired object.
**Z initial speed**	5	Travel speed of the printing plate along the Z axis for the first 30 layers.	. If decreased, the printing time is increased, but it guarantees a more solid base for the object.
**PM initial speed**	15	Tilt speed of the vat for the first 30 layers.	. If decreased, the printing time is increased, but it guarantees a more solid base for the object.

Table S3 - Optimized internal parameters in order to increase the printing speed while leaving untouched the good printing resolution and structural stability.

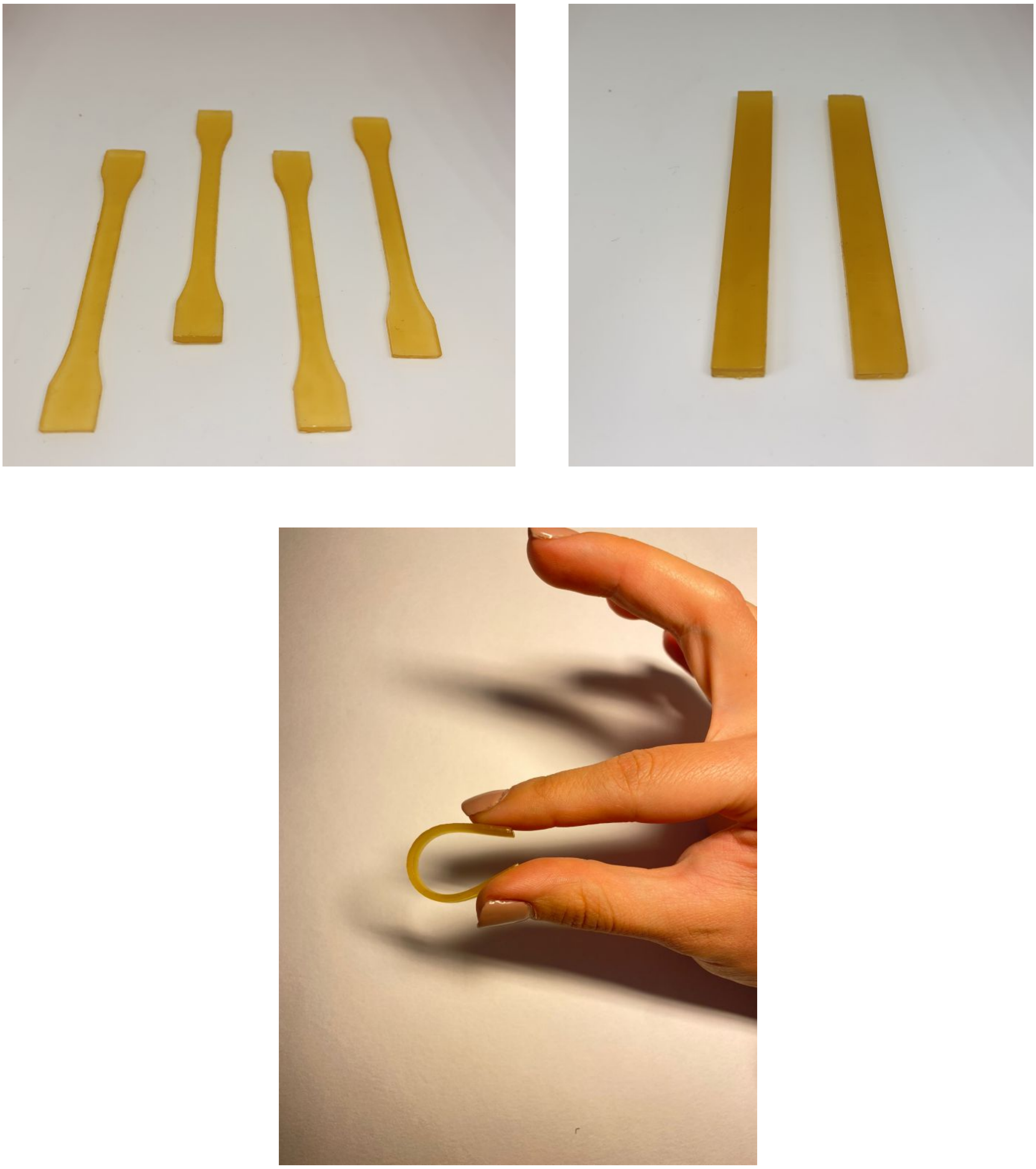

Figure S6 – 3D-Printed specimens and demonstration of their flexibility.
